# ALERT: A benchmark Bengali dataset for identifying and categorizing religiously aggressive texts

**DOI:** 10.1016/j.dib.2025.112094

**Published:** 2025-09-19

**Authors:** Suhana Binta Rashid, Bibhas Roy Chowdhury Piyas, Sadia Rahman, Bijoy Roy Chowdhury Preenon

**Affiliations:** a Department of Computer Science and Engineering, Chittagong University of Engineering and Technology, Chattogram 4349, Bangladesh; b Department of Software Engineering, Daffodil International University, Dhaka 1216, Bangladesh; c Department of Electrical and Electronic Engineering, Independent University, Dhaka 1229, Bangladesh

**Keywords:** Natural language processing (NLP), Machine learning, Deep learning, Bengali religious aggression detection, cybersecurity

## Abstract

The widespread proliferation of religiously aggressive contents on social media platforms poses significant threats to societal harmony and communal solidarity. It often incites religious animosity, provokes violence and disseminates life-threatening messages that intensifies societal divisions and undermines social harmony. Despite significant advancements in identifying such contents in high-resource languages like English, there exists a notable scarcity of resources for regional languages like Bengali which constrains the development of effective detection and prevention tools. To address this gap, we introduce ALERT (Analysis of Linguistic Extremism in Religious Texts), a newly developed Bengali dataset along with English translation which includes 4027 annotated instances classified into four categories: hate speech (995), vandalism (909), atrocity (1117), and no aggression (1006). The dataset was sourced from many online platforms, including Facebook, YouTube, online news websites, blogs and group chats. Each of the instances in the dataset was annotated by any two annotators from the list of four having diverse religious, ethnic, geographical, and academic backgrounds. Any conflicts or disagreements between annotators during the annotation process were resolved through consultation with a domain expert. The preprocessing stages include the elimination of English words, duplication and alphanumeric characters to ensure data integrity. The dataset attains a Cohen’s kappa score of 72 % that signifies a strong inter-annotator agreement and a Jaccard similarity score between 16 % and 23 % which reflects the degree of overlap between classes. Moreover, Experiments with various machine learning, deep learning and transformer-based models yield promising results. ALERT serves as a benchmark dataset for religiously aggressive text classification that may contribute to the advancement of research in this field. The dataset is publicly accessible for research purposes to promote innovation and collaboration within the Bengali NLP community.

Specifications Table

**Table:** 

Subject	Computer Science
Specific subject area	*Machine Learning, Natural Language Processing, Bengali Text Classification, Bengali Religious Aggression Detection*
Type of data	*Text Files (xlsx-formatted)*
Data collection	*Our developed dataset contains 4027 instances collected from multiple online platforms, such as Facebook, YouTube, news websites, Bangla blogs, online forums and group conversations. Each instance was classified into one of four categories: hate speech (995), vandalism (909), atrocity (1117), and no aggression (1006). Each of the instances in the dataset was annotated by any two annotators from the list of four with varied academic, religious, and racial backgrounds. Any conflict was resolved by a subject matter expert to guarantee consistency. To augment reliability, native Bengali speakers meticulously examined the dataset that made it a valuable asset to Bengali NLP research.*
Data source location	*The texts were gathered from a variety of online sources, such as Facebook, YouTube, news websites, Bangla blogs, online forums and group conversations.*
Data accessibility	Repository name: Mendeley Data Data identification number: 10.17632/f4xz5d4fzd.1 Direct URL to data: https://data.mendeley.com/datasets/f4xz5d4fzd/1
Related research article	None

## 1 Value of the Data

• This is the first publicly accessible dataset in the low-resource language Bengali that addresses various levels of religious aggression in texts, including hate speech, vandalism, atrocity, and no-aggression that aims to prevent the proliferation of religiously aggressive contents on social media.
• The dataset was collected from prevalent online platforms like Facebook, YouTube, news websites, Bengali blogs, online forums, and group conversations that encapsulates real-world linguistic, cultural, religious, and emotional variations which made it rich and diverse in the context of religious hostility.
• It aids the development of robust and context-aware models to identify religiously detrimental contents by utilising the complex patterns of religious aggression present in the dataset which can enhance digital safety through development of superior content moderation tools.
• The dataset sets a benchmark for detecting religious aggression in Bengali texts that can enhance online safety and mitigate societal conflict. Moreover, it encourages linguists and language researchers to explore further research in this area.

## 2 Background

While recent research has explored various forms of aggression across multiple languages, a notable gap remains in datasets that specifically address the severity levels of religious aggression. Rashid et al. [1] introduced the Bangla Aggressive Dataset (BAD) from social media texts and annotated with both binary (aggressive/non-aggressive) and multiclass labels (religious, political, verbal and gendered). In a related area [2], proposed a multimodal dataset addressing misogynistic content in memes classified as misogynistic or non-misogynistic. Study [3] presented a dataset with annotated tweets in mixed Wolof-French for detecting abusive messages. Study [4] developed an English-language dataset for hate speech detection, while [5] contributed with the HS-BAN dataset for binary hate speech classification in Bengali. Das et al. [6] further expanded on hate categorization with a dataset curated from Bengali social media comments across seven categories of hate speech [7]. focused on cyberbullying detection in Bengali using data from various platforms. Addressing visual context [8], developed a multimodal dataset combining symbolic images with text and annotated into non-aggressive, medium, and highly aggressive classes. Lastly [9], introduced a dataset for political aggression in Bengali texts with binary and multiclass classification. Hence, there remains a gap in datasets to address religious aggression.

## 3 Data Description

Religion significantly influences societies, identities and social systems. Currently, social media platforms such as Facebook, YouTube, and Twitter serve as common platforms for discussion on religion that enable individuals to rapidly communicate their perspectives on worldwide incidents. With the increasing number of social media users, religious debates frequently arise. Conflicting perspectives can often lead to enmity, hatred, violence that damages the unity of society. This shows the necessity of monitoring online interactions to detect harmful activity and mitigate tensions among various groups.

This study introduces the **ALERT(Analysis of Linguistic Extremism in Religious Text)** dataset, a Bengali corpus focused on religiously aggressive texts. We put together a diverse dataset that reflects different levels of religious aggression, each showing its own unique language patterns. To make things clear, we carefully defined each category to facilitate accurate understanding and annotation.

1. **Hate Speech**: Text that expresses intolerance, prejudice, hostility, or discriminatory attitudes against a religion, its symbols, practices, or followers. This includes derogatory remarks, stereotyping, incitement of animosity, or any language promoting religious bias.
2. **Vandalism**: Text describing acts of intentional damage, destruction, defacement, or desecration of religious property, sites, symbols, or objects motivated by religious hostility or hatred. This includes attacks on temples, mosques, churches, statues, scriptures, or other religious artifacts.
3. **Atrocity:** Text describing severe and cruel acts of violence or oppression motivated by religious hatred or targeting religious groups. Examples include murder, torture, mass killings, death, or other forms of large-scale violent persecution explicitly linked to religious motives.
4. **Non-Aggressive**: Text that is respectful, peaceful, and promotes harmony toward all religions, their symbols, practices, and followers. It contains no hostility, intolerance, prejudice, or derogatory language related to any religion or religious group.

**Fig. 1:**
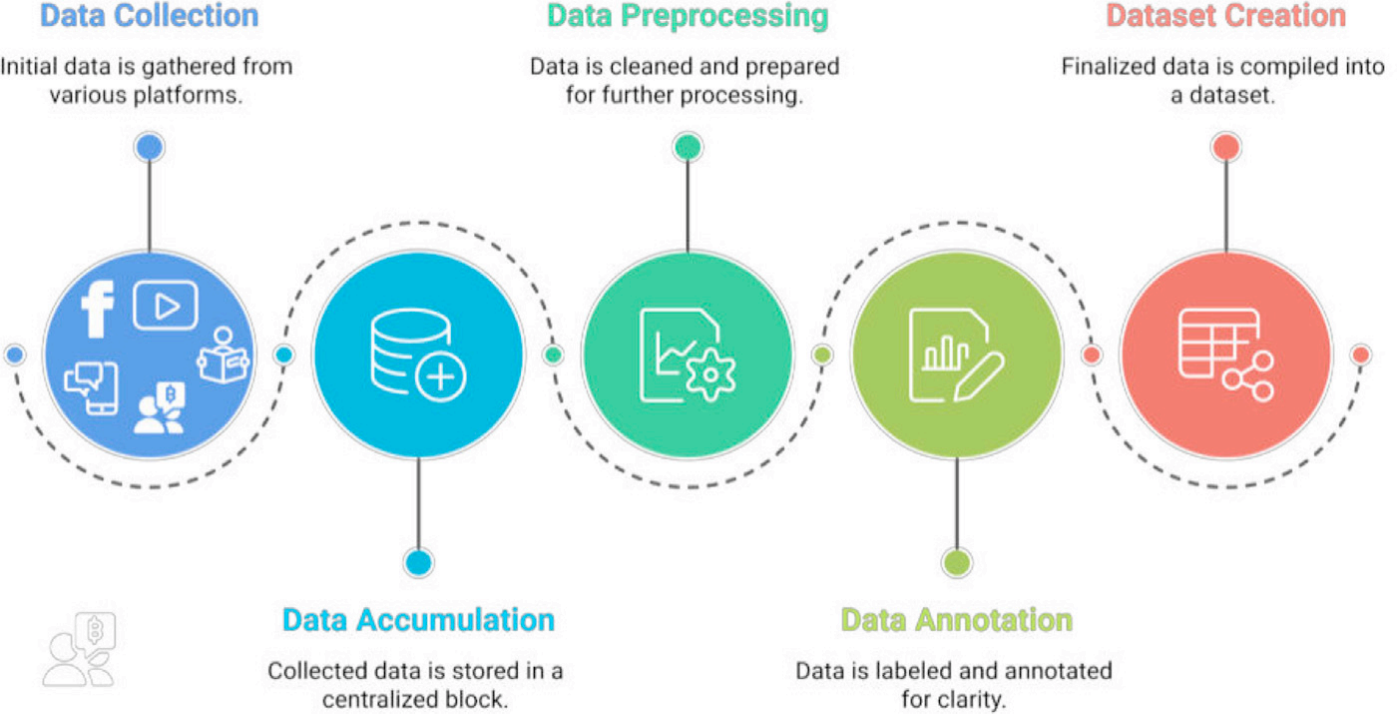
Data collection to dataset creation workflow.

We created the ALERT dataset using established data development methods presented in Fig. 1.

In this section, we discuss data acquisition, the annotation procedure, calculation of annotation agreement, and dataset analysis to provide deeper insights into our developed 'ALERT' dataset.

### 3.1 Dataset acquisition

**Fig. 2:**
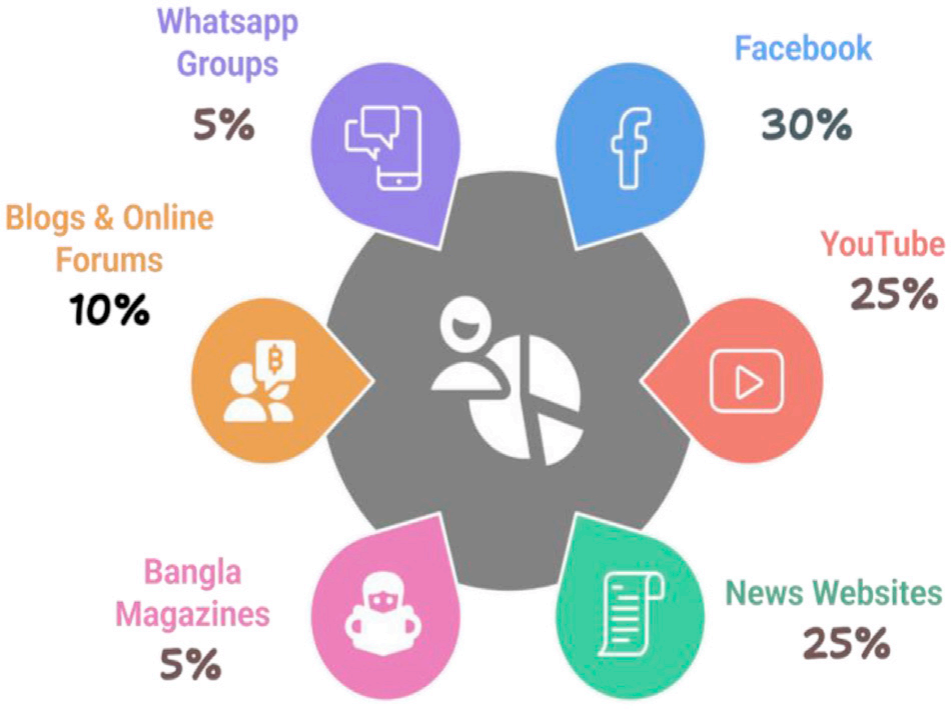
Data collection sources.

The dataset employed in this research was gathered from several online platforms, including Facebook, YouTube, news websites, blogs and online news websites. We excluded Twitter due to its limited availability of Bengali content. Recent social media data indicate that 30.4 % of social media users in Bangladesh actively use Facebook, whereas 19.3 % connected with YouTube. Thus, a significant portion of the dataset samples was obtained from these two platforms, as they have the highest concentration of Bengali social media users. A focused methodology was employed to identify religiously aggressive contents by paying special attention on social media posts, comments, and replies. Verified social media accounts associated with religious scholars, their supporters, and opposing groups were closely monitored due to their influential role in shaping public opinion. Furthermore, notable occurrences of assaults on religious minorities and disputed events during religious festivals were meticulously examined to identify aggressive content. Content for non-aggressive text data was sourced from Facebook and YouTube posts and comments, in addition to news websites, blogs and discussion forums. This included materials shared by religious institutions, scholars and community groups from both majority and minority communities. Only conversation threads with at least 200 reactions were considered relevant and engaging. Fig. 2 illustrates the sources of data, while Table 1 presents the overall status of the data collection sources.

**Table 1:** Statistical information about the data collection source.

Name	Facebook	Online News Portal	YouTube	Bangla Magazines	Online Blogs	WhatsApp Group
**Affiliation**	Social Media	News Platform	Video Platform	Digital Publication	Digital content	Messaging Platform
**Popularity**	2.8 B	1.5 M	2. 3 B	200,000	500,000	Varies (depends on group size)
**Activity**	50–100 posts/day	20–30 posts/day	500 videos/day	3–5 posts/day	10–15 posts/day	Frequent (varies per group)

### 3.2 Dataset visualization

**Fig. 3:**
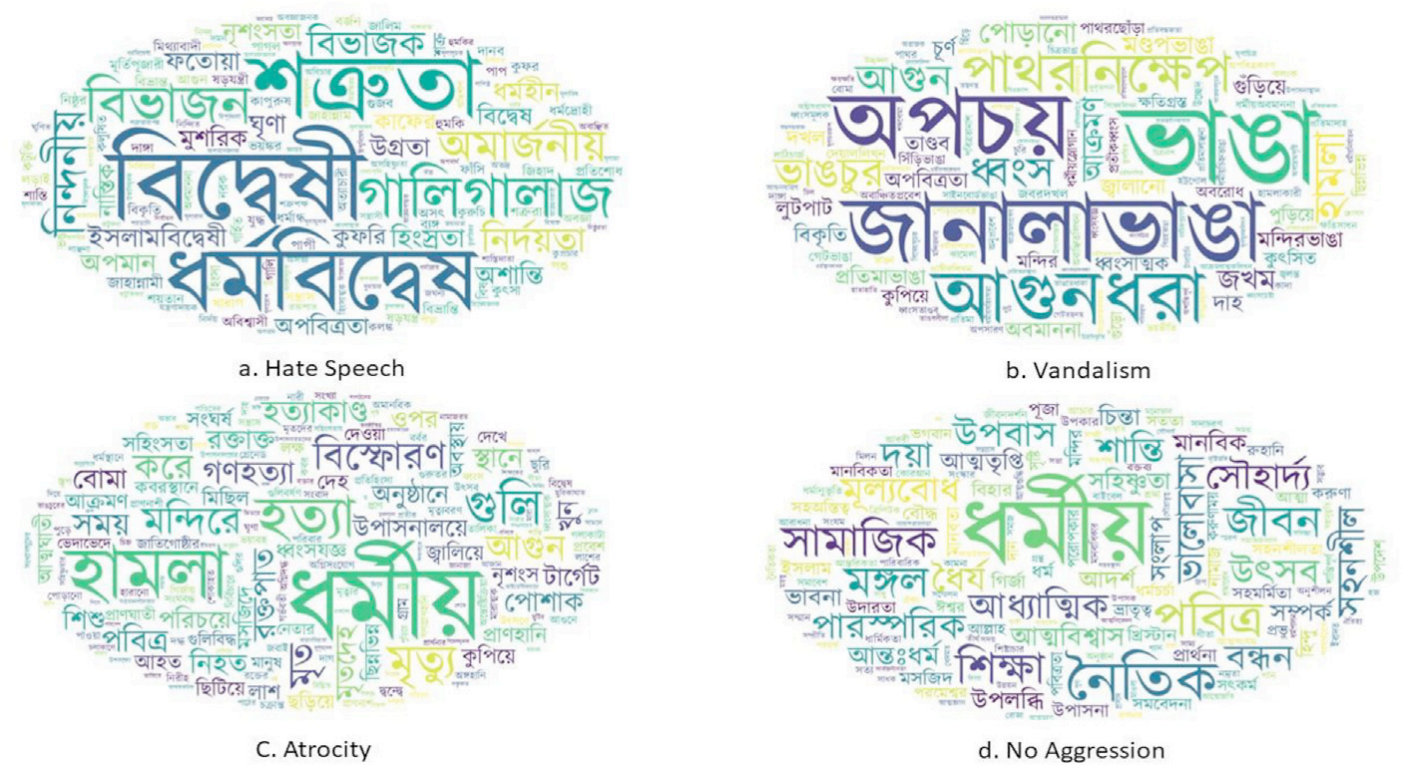
Word clouds for different Aggression classes using Bengali dataset.

**Fig. 4:**
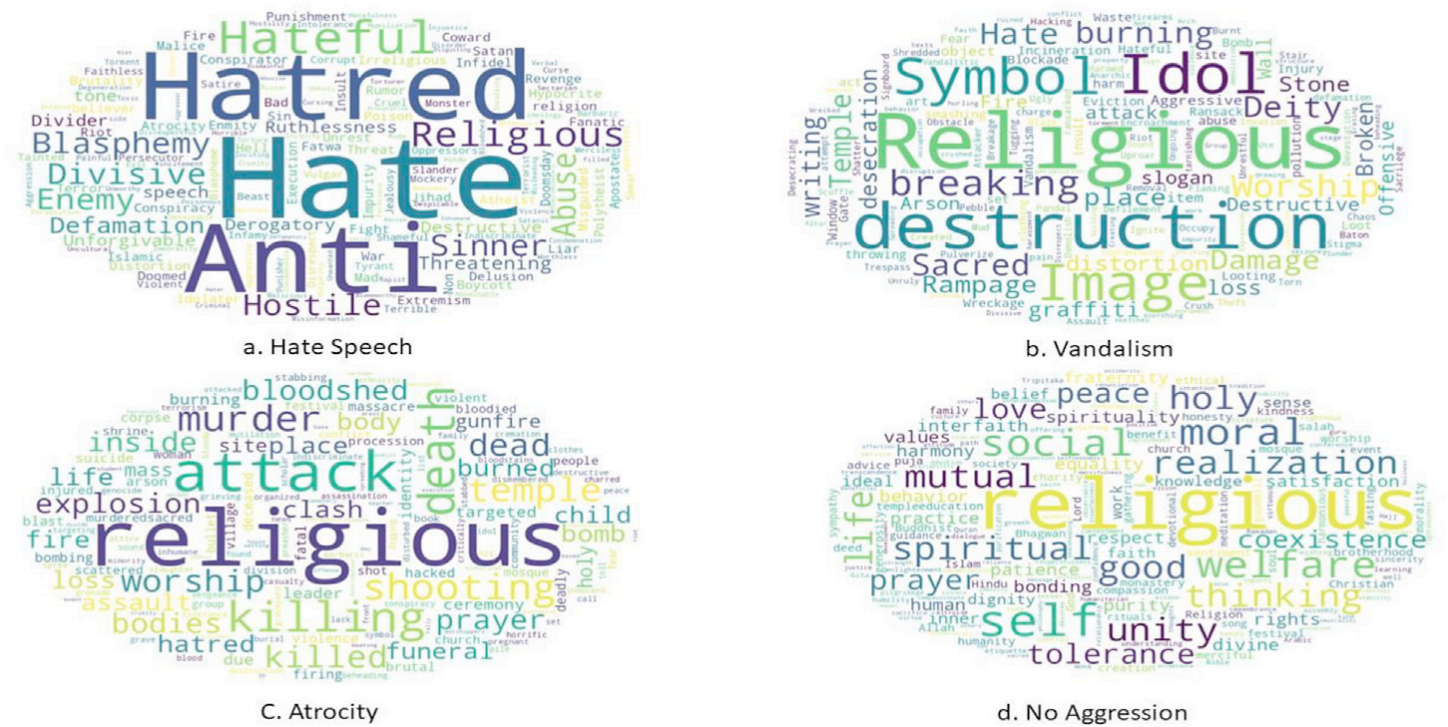
Word clouds for different aggression classes using the English-translated dataset.

To visualize a text dataset, word cloud is crucial for highlighting the most frequent words in each class that can offer valuable insights into the linguistic patterns within the dataset. Additionally, identifying the top words that significantly contribute to classifying an instance can serve as an effective metric for data visualization. Fig. 3, Fig. 4 illustrate word clouds representing the 200 most frequent words from each class in the Bengali and English-translated datasets respectively, while Table 2 presents the most significant words from each of the classes.

**Table 2:** Top words with English meanings for each class.

A few instances from the ALERT dataset with their final annotations are presented in Table 3.

**Table 3:** Sample text entries with their respective categories in ALERT dataset.

### 3.3 Dataset analysis

**Fig. 5:**
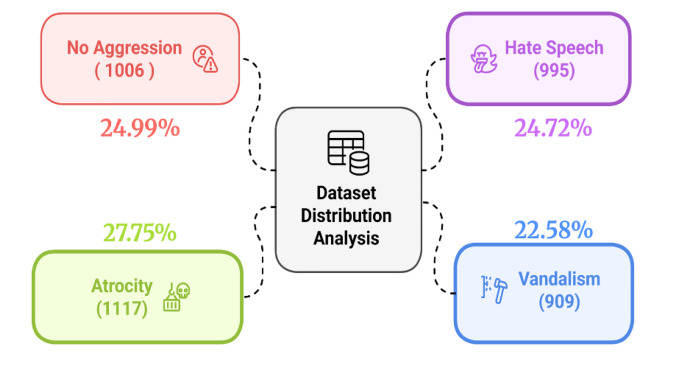
Analysis of dataset distribution with sample counts.

Fig. 5 presents the dataset distribution that shows the number of instances in each class along with their corresponding percentages within the overall dataset. A detailed statistical analysis of the dataset shown in Table 4 that reveals key insights for model development. The lexical analysis shows clear variations in word count and diversity across categories. Atrocity has the highest total word count (26,111) and highest average text length (23.38), while Hate speech has the lowest. No aggression is typically found in medium-length texts, suggesting that religiously provocative content tends to be either too short or too long. On the other hand, atrocity texts are generally more detailed, as they often convey statistical information such as the number of deaths and losses. These findings indicate that the volume of text individuals write may fluctuate based on the nature of hostility in the communication.

**Table 4:** Summary of text statistics across different classes.

Class Name	Total words	MTL (words)	ANW (per text)	Unique words	ANUW (per text)
**Hate Speech**	20,629	66	20.73	6385	19.74
**Vandalism**	21,150	85	23.27	6058	22.14
**Atrocity**	26,111	73	23.38	5422	22.49
**No Aggression**	22,150	88	22.02	6644	20.66

MTL = Maximum Text Length, ANW = Average Number of Words per Text, ANUW = Average Number of Unique Words per Text.

In Table 5, the Jaccard similarity analysis reveals the highest similarity (0.23) between the Vandalism and Atrocity categories, while the lowest similarity (0.16) is found between No Aggression and Vandalism.

**Table 5:** Jaccard similarity value for each class.

Class Name	Hate Speech	Vandalism	Atrocity	No-Aggression
**Hate Speech**	-	0.19	0.18	0.21
**Vandalism**	-	-	0.23	0.16
**Atrocity**	-	-	-	0.17
**No Aggression**	-	-	-	-

**Fig. 6:**
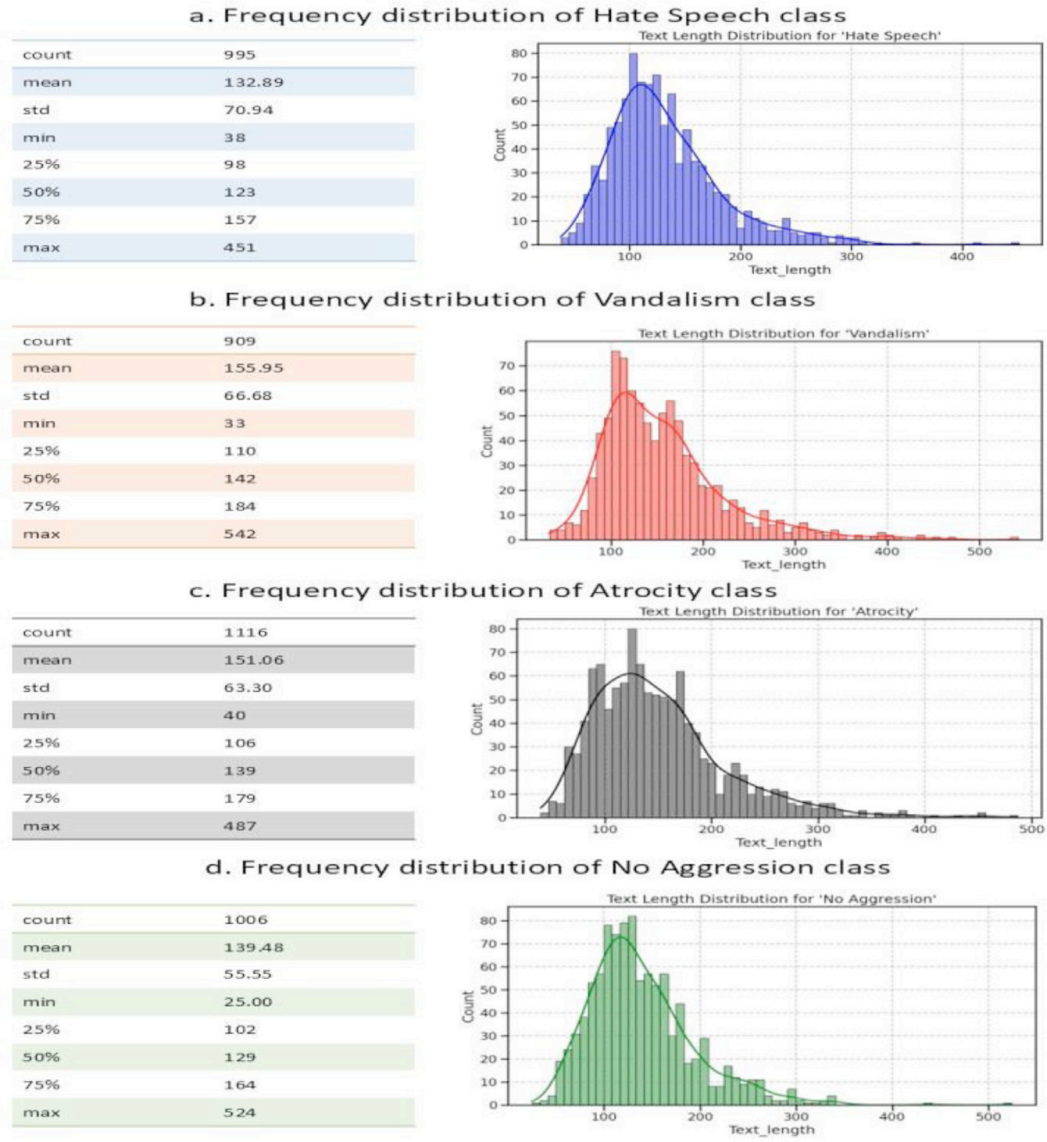
Text length distribution for Hate Speech, Vandalism, Atrocity and No Aggression classes.

Fig. 6 shows the frequency distribution of text lengths across the four classes in the dataset. The **Hate Speech** class includes 995 instances with an average length of 132.89 characters where most of the texts fall between 98 and 157 characters, with a maximum of 451. The **Vandalism** class, consisting of 909 samples, has the highest average length at 155.95 characters and displays a broader distribution with lengths ranging from 33 to 542 characters. The **Atrocity** class is the largest, with 1116 instances and an average length of 151.06 characters, reaching up to 451 characters. In contrast, the **No Aggression** class contains 1006 samples and shows the shortest mean length of 139.48 characters along with the lowest standard deviation 55.55 which indicates more consistent and tightly clustered text lengths compared to the other categories.

While recent research has explored various forms of aggression across multiple languages, to the best of our knowledge, no existing dataset specifically addresses the varying severity levels of religious aggression. However, in the broader domain of aggression detection and related areas including political aggression, misogynistic aggression, hate speech and toxic content, numerous datasets have been developed over the years. Table 6 presents a comparative analysis of these existing datasets with ours to highlight both the contextual similarities and differences between our developed dataset and prior work.

**Table 6:** Comparison with existing work.

Ref	Dataset Name	Context	Label Type	Language	Volume	Year
[1]	BAD	Aggression Detection	Binary and Multiclass	Bangla	14,158	2022
[2]	–	Misogynistic content detection	Binary	English	800	2022
[3]	AWOFRO	Abusive text detection	Binary	Wolof & French	3500	2025
[4]	–	Hate Speech Detection	Binary	English	451,709	2023
[5]	HS-BAN	Hate Speech Detection	Binary	Bangla	50,000	2021
[6]	–	Hate Speech Detection	Multiclass	Bangla	5600	2021
[7]	–	Cyberbullying Detection	Multiclass	Bangla	12,282	2024
[8]	MMHS150K	Aggression Detection	Multiclass	Bangla	7425	2021
[9]	–	Political Aggression Detection	Binary & Multiclass	Bangla	3056	2023
**Proposed**	**ALERT**	**Religious Aggression Detection**	**Multiclass**	**Bangla**	**4027**	**–**

## 4 Experimental Design, Materials and Methods

**Fig. 7:**
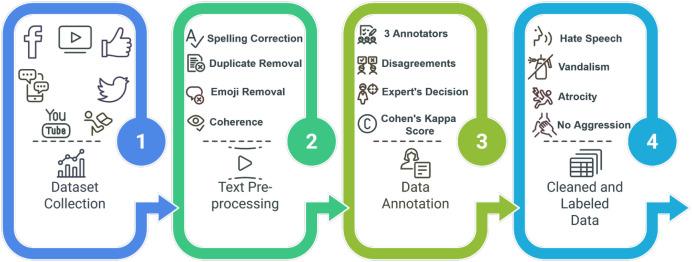
Workflow diagram of dataset generation process.

This section describes the overall experimental design, including the methods used for data collection, preprocessing and annotation in the development of the dataset. Fig. 7 presents the structured workflow of the dataset generation process, showing each stage from initial data collection to the final labeling phase.

### 4.1 Preprocessing

Raw datasets frequently contain inconsistencies that may hinder effective model training. To guarantee the quality and reliability of the ALERT dataset, multiple preprocessing steps were implemented following data collection. This included spelling correction, emoji removal, redundancy removal and the assurance of sentence coherence. English words and unnecessary symbols were removed from dataset to reduce data complexity. English words that do not relate to religious aggression and only introduce noise were removed, while words directly connected to religious aggression were kept in the dataset. Additionally, some words were anonymized to mitigate sensitivity and maintain the dataset's integrity. To maintain coherence, we organized the words logically and used consistent terminology throughout the text.

### 4.2 Data annotation

**Fig. 8:**
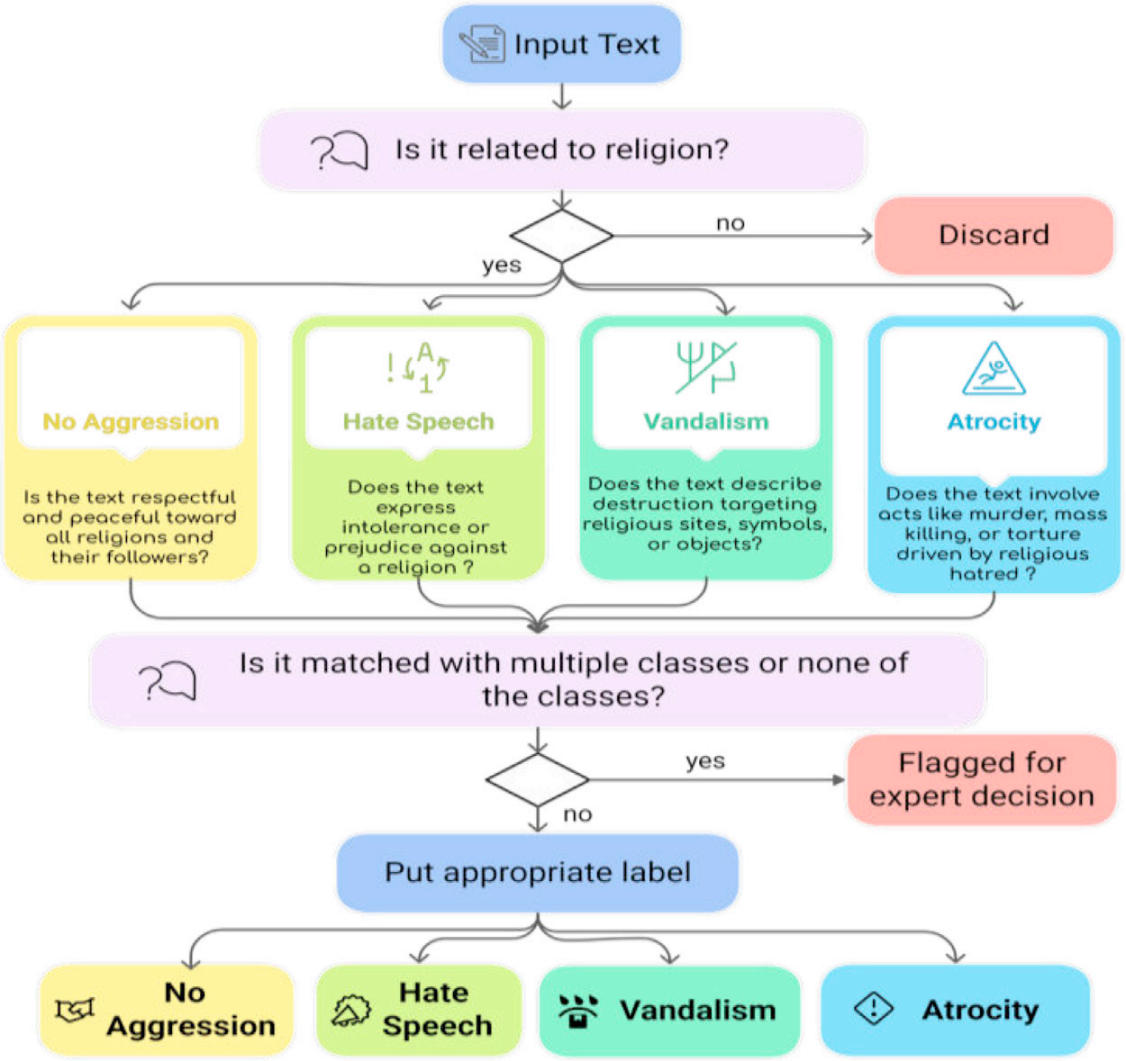
Dataset annotation criteria.

Each data instance was subsequently annotated by two annotators chosen from a pool of four based on their availability and workload distribution, with disagreements resolved by a domain expert. To mitigate bias, annotators were selected from diverse academic, gender, religious, and ethnic backgrounds. To mitigate inconsistencies during the annotation process, all annotators were provided with annotation guidelines, as shown in Fig. 3, to label instances based on specific questions. Additionally, before starting the annotation, each annotator was given a set of example instances along with explanations behind the labels which helped make the process smoother. Fig. 8 illustrates the structured workflow of the dataset generation process demonstrating each stage from initial data collection to the final labelling phase.

### 4.3 Annotation procedure

To address potential biases, annotators with diverse academic, cultural, racial and religious backgrounds were selected. The manual annotation was performed by five native Bengali speakers and their details summarized in Table 7.

**Table 7:** Information of annotators.

	Academic Level	Area of Study	Research Experience	Age	Gender	Religion	Targeted by ORA
An-1	Undergraduate	NLP	2	23	Female	Islam	Yes
An-2	Graduate	NLP	2	25	Male	Islam	No
An-3	Graduate	NLP	2.5	25	Female	Hindu	Yes
An-4	Graduate RA	NLP	3	27	Male	Hindu	Yes
Expert	Assistant Professor	NLP, Cybersecurity	9	33	Male	Islam	No

An = Annotator, ORA = Online Religious Aggression.

To ensure accurate annotation, it is essential to establish explicit annotation criteria as individuals' ways of thinking may differ. Annotators must consider specific guiding questions to correctly assign a class to each instance. Without well-defined criteria, ambiguity and inconsistency may arise that can lead to unreliable results. A well-defined annotation criterion that we followed is illustrated in Fig. 8.

In this dataset each of the instances was annotated by any two annotators selected from a list of four, with conflicts resolved through domain expert judgement. Instances with unclear messages or overlaps are assigned for additional discussion with domain expert. Algorithm 1 shows the overall annotation guidelines to prepare the dataset.

**Algorithm 1:** Step-by-step process for data annotation.

**Input**: Set of texts without labels
**Output:** Annotated Religious aggression text

1. T ← {t_1_, t_2_, …, t_n_} (set of accumulated texts)
2. RA ← [] (Religious aggression text dataset)
3. D_disagree ← [] (Separate list for texts with annotator disagreement)
4. for t_i_ ∈ T do
5. l_1_ ← Annotator 1 assigns label to t_i_
6. l_2_ ← Annotator 2 assigns label to t_i_
7. if l_1_ == flag and l_2_ == flag then
8. Discard text
9. else if l_1_ == l_2_ then
10. RA.append(t_i_, l_1_)
11. else
12. D_disagree.append(t_i_)
13. end if
14. i ← *i* + 1
15. end for
16. for d_j_ ∈ D_disagree do
17. 1. Expert discuss with annotators:
18. 2. Either add d_j_ to 'RA' with final annotation or discard it;
19. j ← *j* + 1
20. end for

### 4.4 Calculation of annotators agreement

a) The 'ALERT' dataset was analysed and annotated by two independent annotators and any disagreements between them during the annotation process were resolved by a domain expert. To assess the level of agreement, we calculated Cohen’s kappa coefficient, as outlined in Eq. (1).
  (1) $$K=\frac{P_{o}-P_{e}}{1-P_{e}}$$
b) Here, P_o_ denotes the actual level of agreement observed among annotators, whereas P_e_ indicates the expected agreement that might occur by chance. Table 8 presents the kappa scores, with the highest agreement (0.82) for the Atrocity category and the lowest (0.61) for Hate Speech. Overall, the average kappa score of 0.72 reflects strong consistency between annotators.

**Table 8:** Cohen's Kappa value for each class.

Class Name	Kappa Score	Mean Score
Hate Speech	0.61	0.72
Vandalism	0.76	
Atrocity	0.82	
No Aggression	0.71

### 4.5 Examples of annotation agreements and disagreements

a) Table 9 presents few instances where two annotators provided differing opinions, which were further discussed with a domain expert to resolve the conflicts and finalize the annotations.

**Table 9:** Example texts with annotator disagreements and expert remarks.

### 4.6 Ablation study

To ensure that the preprocessing decisions applied during dataset construction were well-justified rather than arbitrary, we conducted a small ablation analysis. Specifically, we examined the impact of modifying certain preprocessing steps on the dataset’s characteristics. Key operations included the removal of emojis, special characters, English tokens as well as the anonymization of user mentions and named entities. We observed that removing unnecessary English tokens reduced the proportion of code-mixed text, while the removal of emojis and special characters lowered noise and reduced the overall vocabulary size. Anonymization, in turn, sharply decreased the occurrence of identifiable names, ensuring privacy protection. Alongside descriptive statistics, we also trained a lightweight baseline model on both the raw and pre-processed datasets to illustrate the effects of these steps. The results confirmed that removing unnecessary characters, emojis, and English words produced a cleaner dataset, while anonymization effectively safeguarded data privacy with negligible impact on dataset utility.

## Limitations

The ALERT dataset offers valuable insights though it is not without certain limitations. The dataset can be enhanced by incorporating a broader range of aggression types. Also, more complex forms of expressions like mixed aggression and paradoxical sentiments can be incorporated to ensure the robustness of the dataset. Moreover, the use of mixed-code language, such as Bengali-English and language switching, is increasingly common in everyday conversation. The enhanced version of the dataset will account for this by incorporating more such sentences.

## Ethics Statement

The ALERT dataset has been developed on ethical data collection principles. All content was cautiously obtained from publicly accessible sources, including blogs, Facebook pages, news websites and blogs. To mitigate the possibility of copyright infringement, strict selection procedures were carried out to guarantee the inclusion of only copyright-free material. The dataset adheres to responsible use principles, emphasising the safeguarding of individual rights and the prevention of harm. Given the sensitive nature of the topic, significant care was taken to avoid targeting or harming any particular country, religion, or community. To ensure fairness and neutrality, the dataset was independently reviewed by a group of volunteers who assessed the content for potential bias or harm. Our primary goal is to support the development of content moderation tools aimed at reducing religious aggression online. This work is not intended to criticize or demean any religion or belief system. Data obtained from Facebook sites adhered to Facebook's content restrictions, negating the necessity for further authorisation. We would also like to express our sincere gratitude to the volunteers who reviewed the dataset to help mitigate potential bias, harm, and inconsistencies.

## Credit Author Statement

**Suhana Binta Rashid:** Conceptualization, Data curation, Methodology, Software, Writing – original draft, Visualization. **Bibhas Roy Chowdhury Piyas:** Conceptualization, Data curation, Methodology, Software, Visualization, Writing – review & editing. **Sadia Rahman:** Data curation, Investigation, Validation, Writing – review & editing. **Bijoy Roy Chowdhury Preenon:** Data curation, Validation, Writing – review & editing.

## Data Availability

Mendeley DataALERT: A Benchmark Dataset for Detecting Religious Aggression in Bengali Texts (Original data).
